# DNMT3B silencing suppresses migration and invasion by epigenetically promoting miR-34a in bladder cancer

**DOI:** 10.18632/aging.103820

**Published:** 2020-11-20

**Authors:** Kai Xu, Binshen Chen, Bingkun Li, Chaoming Li, Yiming Zhang, Ning Jiang, Bin Lang

**Affiliations:** 1 Department of Urology, Zhujiang Hospital of Southern Medical University, Guangzhou, People’s Republic of China; 2 Guangzhou Key Laboratory of Inflammatory and Immune Diseases, Zhujiang Hospital of Southern Medical University, Guangzhou, People’s Republic of China; 3 School of Health Sciences, Macao Polytechnic Institute, Macao, People's Republic of China

**Keywords:** DNA methyltransferase 3B, metastasis, miR-34a promotor, methylation, bladder cancer

## Abstract

The role of DNA methyltransferase 3B (DNMT3B) in tumorigenesis and development has been widely recognized; however, the mechanism underlying its action remains unclear. Considering its function in *de novo* methylation, we aimed to investigate whether DNMT3B plays its role via microRNA (miR)-34a promoter methylation in bladder cancer. We found that DNMT3B expression was low in 10 bladder cancer tissues and high in 20 bladder cancer tissues. miR-34a expression was higher in bladder cancer tissues with low expression of DNMT3B than that in bladder cancer tissues with high expression of DNMT3B. The level of miR-34a was negatively correlated with the level of DNMT3B. The methylation ratio of the miR-34a promoter was positively correlated with the level of DNMT3B and negatively correlated with the level of miR-34a. *DNMT3B* knockdown increased the expression of miR-34a and the transcriptional activity of the miR-34a promoter, while decreasing miR-34a promoter methylation. *DNMT3B* knockdown inhibited migration and invasion, while decreasing the protein levels of hepatocyte nuclear factor 4 gamma and Notch1 which are downstream targets of miR-34a. These inhibitory effects of DNMT3B were mitigated by the miR-34a inhibitor. In conclusion, DNMT3B silencing suppresses migration and invasion by epigenetically promoting miR-34a in bladder cancer.

## INTRODUCTION

Bladder cancer, a malignant tumor in the bladder mucosa, is the 10^th^ most common type of cancer worldwide [1]. According to global cancer statistics, bladder cancer has an estimated 549,000 new cases and 200,000 deaths in 2018 [1]. Bladder cancer not only affects the functions of the urinary system but is prone to recurrence and metastasis. Currently, the mechanisms of bladder cancer progression and metastasis are unclear. Therefore, it is crucial to identify the key regulators and underlying mechanisms involved in bladder cancer progression and metastasis.

DNA methylation is an important epigenetic modification in regulating gene expression [2], and has been associated with tumor formation and development. DNA methylation is catalyzed by methyltransferases. DNA methyltransferase 3B (DNMT3B) is a member of the DNA methyltransferase family and functions in *de novo* methylation rather than the maintenance of methylation [2]. It is located primarily in the nucleus, and its expression is developmentally regulated. DNMT3B is upregulated in various malignant tumors, such as lung cancer, hepatocellular carcinoma, prostate cancer, melanoma, and bladder cancer. It is widely recognized to be involved in the regulation of tumor initiation and progression [3–9], but the mechanism remains unclear. Due to its function in *de novo* methylation, we hypothesized that DNMT3B might be a key factor that stimulates promoter methylation of the genes involved in bladder cancer progression and metastasis.

MicroRNAs (miRNAs) are noncoding RNAs that are transcribed but not translated into proteins. Instead, they post-transcriptionally regulate other protein-encoding genes by degrading mRNAs or inhibiting their translation. The miR-34 family is a critical regulator of tumorigenesis and development [10–12]. In bladder cancer, miR-34a expression is correlated with tumor stages, and patients with high miR-34a levels experience longer time to delayed recurrence [10]. Low expression of miR-34a was observed in various types of cancers, including pancreatic, colon, lung, and bladder cancers [10–12]. However, the regulators that decrease miR-34a expression are unclear. It is reported that miR-34a expression and its promoter methylation are inversely correlated in ovarian cancer [13], indicating that the promoter methylation may decrease miR-34a levels in cancer cells. Therefore, the identification of the factors that affect miR-34a promoter methylation is important for illustrating the mechanism of miR-34a in bladder cancer.

In this study, we explored the correlation between miR-34a promoter methylation and DNMT3B expression in bladder cancer. We also investigated whether DNMT3B promoted the migration and invasion of bladder cancer by epigenetically suppressing miR-34a. This study enhances our understanding of the mechanisms of DNMT3B and miR-34a in the migration and invasion of bladder cancer.

## RESULTS

### Correlation of DNMT3B expression and clinicopathological characteristics

**Figure 1:**
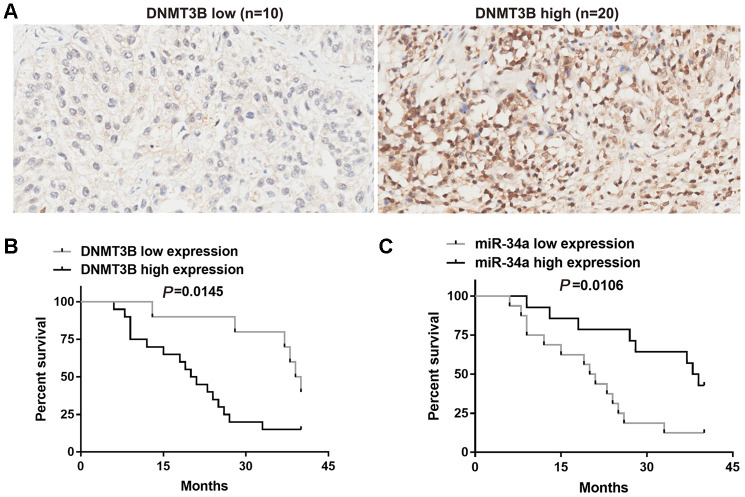
**Correlation between DNMT3B and miR-34a in bladder cancer tissues.** (**A**) The protein level of DNMT3B was assessed using tissue microarrays in 30 bladder cancer tissues. (**B**, **C**) Overall survival analysis of bladder cancer patients who were divided into two groups based on the expression level of DNMT3B (**B**) or miR-34a (**C**). Survival analysis was performed using the Kaplan-Meier method and the logrank test. DNMT3B, DNA methyltransferase 3B.

DNMT3B expression in 30 bladder cancer tissues was assessed using tissue microarrays and scored independently by two senior pathologists. Based on the total scores, DNMT3B was found to be weakly expressed in 10 bladder cancer tissues and highly expressed in 20 bladder cancer tissues (Figure 1A). The association between the expression of DNMT3B and clinicopathological characteristics was analyzed using the Fisher exact test. DNMT3B expression was not correlated with gender (*P* >0.9999), age (*P* = 0.6821), or tumor size (*P*=0.7055), but was correlated with tumor (T) stages (*P*=0.0187), node metastasis (*P* =0.0235), and the muscle-invasive stage (*P* =0.0449) (Table 1).

**Table 1:** Correlation between DNMT3B expression and clinicopathological characteristics.

**Characteristic**	**n=30**	**Expression**		***P***
		**High group (n=20)**	**Low group (n=10)**	
Gender				>0.9999
Male	23	15	8	
Female	7	5	2	
Age				0.6821
<65	8	6	2	
≥65	22	14	8	
Tumor size				0.7055
<5	17	12	5	
≥5	13	8	5	
T stage				0.0187
T1+T2	14	6	8	
T3+T4	16	14	2	
Node metastasis				0.0235
No	18	9	9	
Yes	12	11	1	
Muscle-invasive				0.0449
Yes	10	4	6	
No	20	16	4	

### Survival analysis of bladder cancer patients with different expression of DNMT3B and miR-34a

The bladder cancer patients were divided into two groups by the protein levels of DNMT3B. Overall survival was assessed using the Kaplan-Meier method and the logrank test. The median survival time in the group with low DNMT3B expression (n=10) was 39.5 months (Figure 1B), and that in the group with high DNMT3B expression (n=20) was 20.5 months (Figure 1B). This indicated that patients with lower expression of DNMT3B had a longer overall survival compared to those with higher expression of DNMT3B (Figure 1B). Thereafter, we assessed the level of miR-34a expression in the 30 bladder cancer tissues. Based on the median miR-34a level, bladder cancer tissues were divided into low-miR-34a-expression group and high-miR-34a-expression group. The median survival time in the low-miR-34a-expression group (n=16) was 20.5 months (Figure 1C), and that in the high-miR-34a-expression group (n=14) was 38.5 months (Figure 1C). This indicated that patients with higher expression of miR-34a had longer overall survival compared to those with lower expression of miR-34a (Figure 1C).

### Correlation of DNMT3B and miR-34a levels in bladder cancer tissues

**Figure 2:**
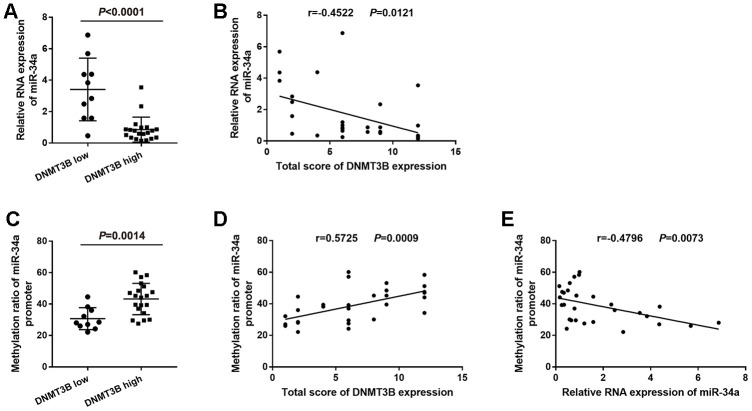
**Correlation between the level of DNMT3B and the methylation ratio of the miR-34a promoter in bladder cancer tissues.** (**A**) Relative RNA expression of miR-34a in low-DNMT3B-expression and high-DNMT3B-expression bladder cancer tissues. (**B**) Correlation between the total score of DNMT3B expression and the relative RNA expression of miR-34a in bladder cancer tissues. (**C**) The methylation ratio of the miR-34a promoter in low-DNMT3B-expression and high-DNMT3B-expression bladder cancer tissues. (**D**) Correlation between the total score of DNMT3B expression and the methylation ratio of the miR-34a promoter in bladder cancer tissues. (**E**) Correlation between the relative RNA expression of miR-34a and the methylation ratio of the miR-34a promoter in bladder cancer tissues. DNMT3B, DNA methyltransferase 3B.

According to the level of DNMT3B protein expression, bladder cancer tissues were divided into the low-DNMT3B-expression group and the high-DNMT3B-expression group. miR-34a expression was higher in bladder cancer tissues with low DNMT3B expression compared to those with high DNMT3B expression (Figure 2A). Furthermore, the level of miR-34a was negatively correlated with the level of DNMT3B based on the linear regression analysis (r=-0.4522, *P*=0.0121, Figure 2B).

### Correlation of the DNMT3B level and methylation ratio of miR-34a promoter in bladder cancer tissues

Bisulfite genomic sequencing **(**BSP) analysis showed that the methylation ratio of the miR-34a promoter was lower in the low-DNMT3B-expression bladder cancer tissues compared to that in the high-DNMT3B-expression tissues (Figure 2C). Furthermore, the methylation ratio of the miR-34a promoter was positively correlated with the level of DNMT3B (r=0.5725, *P*=0.0009, Figure 2D), and was negatively correlated with the level of miR-34a (r=-0.4796, *P*=0.0073, (Figure 2E) based on the linear regression analyses.

### Knockdown of *DNMT3B* increased miR-34a expression and decreased methylation in the promoter of miR-34a

**Figure 3:**
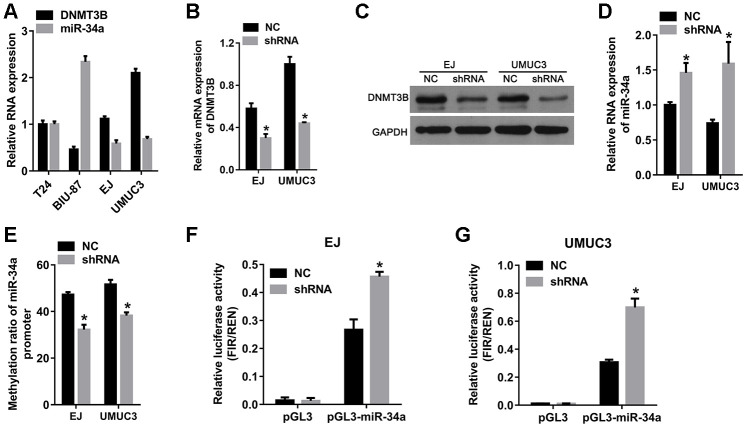
**The knockdown of *DNMT3B* increased miR-34a expression and decreased the methylation of the miR-34a promoter.** EJ and UMUC3 cells were infected with lentiviral vectors expressing shRNAs targeting DNMT3B (shRNA)to construct DNMT3B-knockdown cells, termed as the shRNA group. EJ and UMUC3 cells infected with empty lentivirus were used as the negative control (NC) group. (**A**) Expression of DNMT3B in bladder cancer cells measured by qRT-PCR. (**B**, **C**) Expression of DNMT3B in the NC and shRNA groups as detected by qRT-PCR (**B**) and western blot analysis (**C**). (**D**) The levels of miR-34a in the NC and shRNA groups as detected by qRT-PCR. (**E**) The methylation ratio of the miR-34a promoter as determined by bisulfite genomic sequencing. *DNMT3B* knockdown decreased methylation in the promoter of miR-34a. (**F**–**G**) The effects of *DNMT3B* knockdown on the transcription activity of the miR-34a promoter. (**A**) luciferase reporter plasmid containing the miR-34a promoter (pGL3-miR-34a) was transfected into the shRNA groups of EJ and UMUC3 cells. The empty vector pGL3 was used as NC. The relative luciferase activity was calculated using the ratio of firefly and Renilla luciferase activities. Data were presented as means±SD. *p<0.05 between NC and shRNA. DNMT3B, DNA methyltransferase 3B; qRT-PCR, quantitative reverse transcription polymerase chain reaction; NC, negative control; SD, standard deviation.

Based on the results from bladder cancer tissues, we hypothesized that DNMT3B reduced the expression of miR-34a via miR-34a promoter methylation, which was verified using bladder cancer cell lines. As shown in Figure 3A, the mRNA level of DNMT3B was higher and the level of miR-34a was lower in the EJ and UMUC3 bladder cancer cell lines compared to those in the T24 and BIU-87 bladder cancer cell lines. Therefore, EJ and UMUC3 cells were chosen for subsequent analyses. To further investigate the role of DNMT3B, EJ and UMUC3 cells were infected with lentiviral vectors containing the short hairpin RNA (shRNA) targeting DNMT3B to construct the *DNMT3B*-knockdown cells, termed as the shRNA group. Cells infected by empty lentivirus were used as the negative control (NC) group. As shown in Figure 3B and 3C, the mRNA and protein levels of DNMT3B in the shRNA groups of EJ and UMUC3 cells were decreased compared to those in the NC group, confirming that the *DNMT3B*-knockdown cells were successfully constructed. Thereafter, we examined the miR-34a expression in the *DNMT3B*-knockdown cells. Our data revealed that the miR-34a levels were increased in the shRNA groups of EJ and UMUC3 cells compared to those in the NC groups, indicating that *DNMT3B* knockdown increased miR-34a expression in both EJ and UMUC3 cells (Figure 3D). In addition, the BSP data showed that the methylation ratios of the miR-34a promoter were decreased in the shRNA groups of EJ and UMUC3 cells compared to those in the NC group cells, which indicated that *DNMT3B* knockdown decreased miR-34a promoter methylation in both EJ and UMUC3 cells (Figure 3E). Moreover, we investigated the effect of *DNMT3B* knockdown on the transcriptional activity of the miR-34a promoter. A luciferase reporter plasmid containing the miR-34a promoter (pGL3-miR-34a) was transfected into the NC and shRNA groups of EJ and UMUC3 cells. The empty luciferase reporter plasmid pGL3 was used as a negative control. As shown in Figure 3F and 3G, the relative luciferase activity of pGL3-miR-34a was higher in the shRNA groups of EJ and UMUC3 cells compared to those in the NC group cells, demonstrating that *DNMT3B* knockdown increased the transcriptional activity of the miR-34a promoter in both EJ and UMUC3 cells.

To verify the results in the *DNMT3B-*knockdown cells, *DNMT3B-*overexpression plasmid **(**ov**-**DNMT3B**)** was constructed and transfected into BIU-87 cells to overexpress *DNMT3B*. Empty plasmid pcDNA3.1 was transfected as a negative control. As shown in Supplementary Figure 1A, the DNMT3B protein level was substantially increased in the ov**-**DNMT3B transfected BIU-87 cells compared to that in the pcDNA3.1 transfected cells, confirming the overexpression of *DNMT3B*. The level of miR-34a was decreased (Supplementary Figure 1B), the methylation ratio of the miR-34a promoter was increased (Supplementary Figure 1C), and the relative luciferase activity of pGL3-miR-34a was higher (Supplementary Figure 1D) in the ov**-**DNMT3B transfected BIU-87 cells compared to those in the pcDNA3.1 transfected cells. These results indicated that *DNMT3B* overexpression decreased miR-34a expression, increased miR-34a promoter methylation, and decreased the transcriptional activity of the miR-34a promoter.

### Knockdown of *DNMT3B* inhibits migration, invasion, and epithelial-mesenchymal transition (EMT)

**Figure 4:**
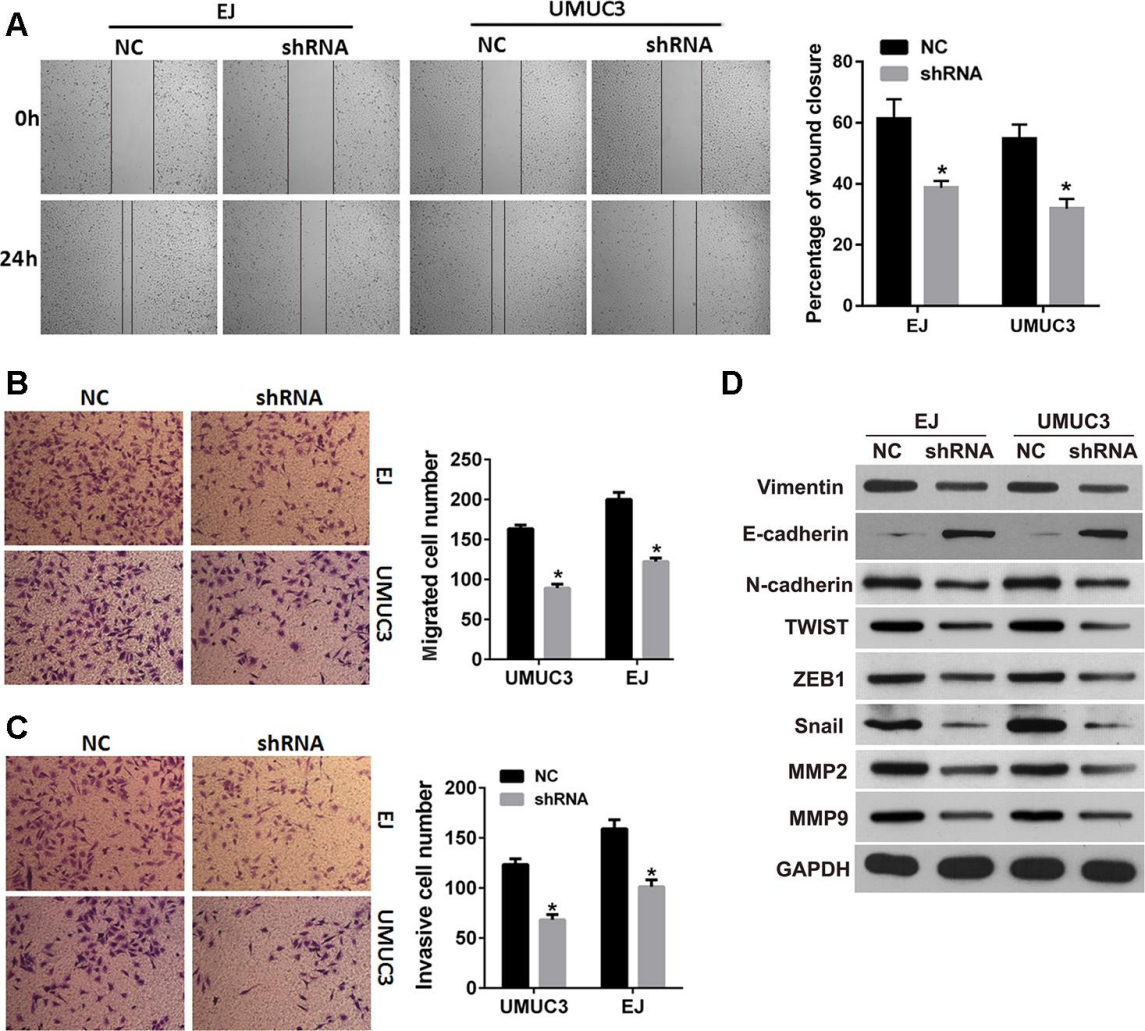
***DNMT3B* knockdown inhibits migration, invasion, and EMT in bladder cancer cells.** EJ and UMUC3 cells were infected with lentiviral vectors expressing shRNAs targeting DNMT3B (shRNA)to construct DNMT3B stable knockdown cells, termed as shRNA group. EJ and UMUC3 cells infected with empty lentivirus were used as the NC group. (**A**, **B**) The wound healing assay (**A**) and the Transwell migration assay (**B**) were performed to evaluate the migration capability. (**C**) The Transwell invasion assay was performed to evaluate the invasion capability. Data were presented as mean±SD. *p<0.05 between NC and shRNA. (**D**) Expression of epithelial-mesenchymal transition markers as detected by western blot analysis. DNMT3B, DNA methyltransferase 3B; EMT, epithelial-mesenchymal transition; NC, negative control; SD, standard deviation.

In order to investigate whether DNMT3B was involved in the migration and invasion of bladder cancer, we evaluated the effects of *DNMT3B* knockdown on the migration, invasion, and EMT in EJ and UMUC3 cells. The results of the wound healing assay revealed that the percentage of wound closure was decreased in the shRNA groups of EJ and UMUC3 cells (Figure 4A) compared to those in the NC groups. The Transwell assays revealed that the numbers of migrated and invasive cells in the shRNA groups of EJ and UMUC3 cells were lower than those of the NC groups (Figure 4B and 4C). These results suggested that *DNMT3B* knockdown resulted in decreased migration and invasion capabilities in EJ and UMUC3 bladder cancer cell lines. Furthermore, the expression of vimentin, N-cadherin, Snail, twist family bHLH transcription factor 1 (TWIST1), zinc finger E-box binding homeobox 1 (ZEB1), matrix metallopeptidase 2 (MMP2), and MMP9 was decreased, and the expression of E-cadherin was increased in the shRNA groups of EJ and UMUC3 cells (Figure 4D). Together, our data indicate that the suppression of *DNMT3B* inhibits EMT in bladder cancer cells.

### Inhibition of miR-34a reduced the effects of *DNMT3B* knockdown on migration, invasion, and EMT

**Figure 5:**
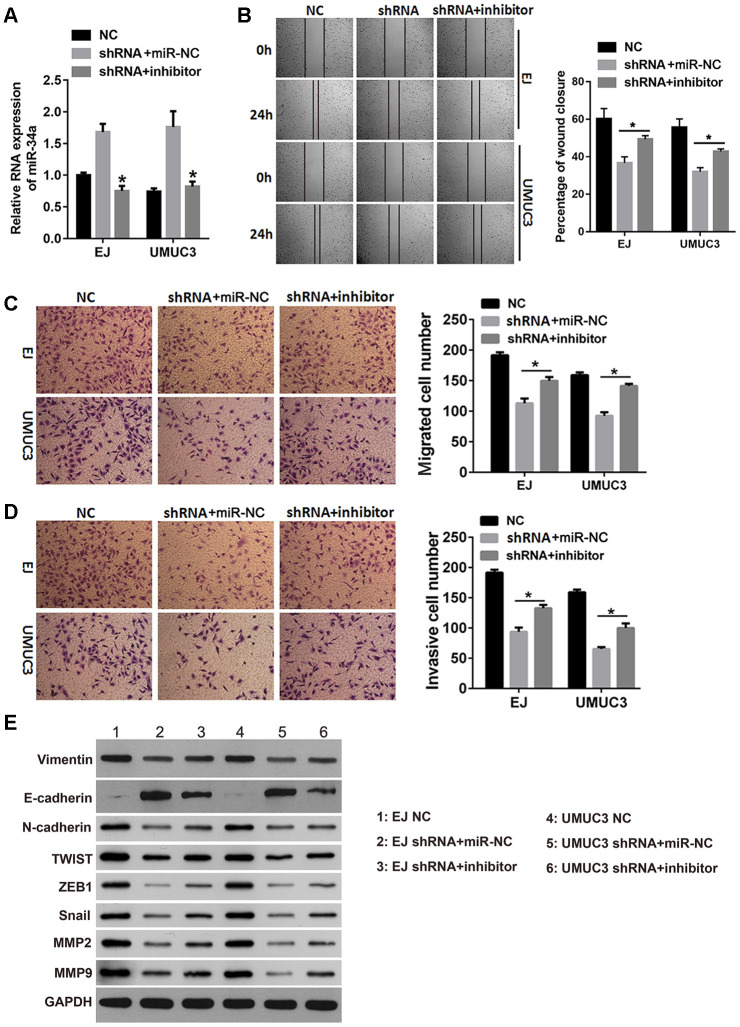
**The miR-34a inhibitor reduced the effects of *DNMT3B* knockdown on migration, invasion, and EMT.** The shRNA groups of EJ and UMUC3 cells were transfected with the miR-34a inhibitor (shRNA+inhibitor) or the negative control miRNA (shRNA+miR-NC). The NC groups were used as the control. (**A**) The miR-34a level was confirmed by qRT-PCR. (**B**, **C**) The wound healing assay (**B**) and the Transwell migration assay (**C**) were performed to evaluate the migration capability of each group of cells. (**D**) The Transwell invasion assay was performed to evaluate the invasion capability of each group of cells. (**E**) The expression of the EMT markers was assessed using western blot analysis, using GAPDH as the loading control. Data were presented as means±SD. *p<0.05 between shRNA+miR-NC and shRNA+inhibitor. DNMT3B, DNA methyltransferase 3B; EMT, epithelial-mesenchymal transition; NC, negative control; qRT-PCR, quantitative reverse transcription polymerase chain reaction; SD, standard deviation.

We next investigated whether inhibiting miR-34a rescued the inhibitory effects of *DNMT3B* knockdown on the migration, invasion, and EMT in bladder cancer cells. After transfecting the miR-34a inhibitor into the shRNA groups of EJ and UMUC3 cells, the miR-34a level was verified by quantitative reverse transcription polymerase chain reaction (qRT-PCR). As shown in Figure 5A, the miR-34a level was lower in the shRNA groups of EJ and UMUC3 cells transfected with the miR-34a inhibitor compared to those transfected with the negative control microRNA (miR-NC), indicating that the miR-34a inhibitor successfully attenuated the effect of DNMT3B knockdown on miR-34a. Additionally, the percentage of wound closure increased in the shRNA groups of EJ and UMUC3 cells transfected with the miR-34a inhibitor compared to those transfected with miR-NC (Figure 5B). The Transwell assays showed increased numbers of migrated and invasive cell in the shRNA groups of EJ and UMUC3 cells transfected with the miR-34a inhibitor compared to those transfected with miR-NC (Figure 5C and 5D). The expression of vimentin, N-cadherin, Snail, TWIST1, ZEB1, MMP2, and MMP9 was increased, and the expression of E-cadherin was decreased in the shRNA groups of EJ and UMUC3 cells transfected with the miR-34a inhibitor, compared to those transfected with miR-NC (Figure 5E). These results reveal that inhibiting miR-34a reduced the inhibitory effects of *DNMT3B* knockdown on migration, invasion, and EMT.

### Effects of *DNMT3B* knockdown on the targets of miR-34a

**Figure 6:**
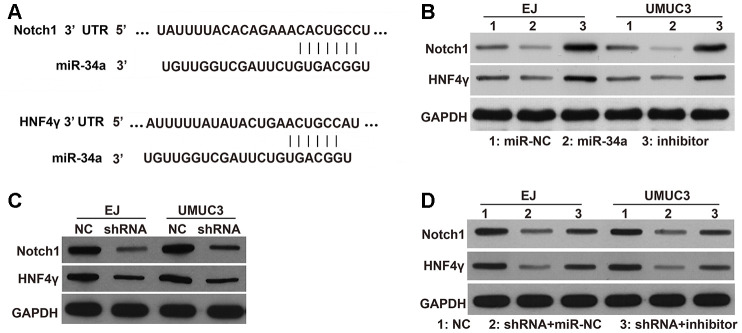
**Effects of *DNMT3B* knockdown on the protein levels of HNF4γ and Notch1, which are downstream targets of miR-34a.** (**A**) miR-34a has binding sites on the 3’ UTR of HNF4γ and Notch1 mRNAs. (**B**) The protein levels of HNF4γ and Notch1 in EJ and UMUC3 cells transfected with miR-NC, miR-34a mimic, or the miR-34a inhibitor (inhibitor). (**C**) The protein levels of HNF4γ and Notch1 in *DNMT3B*-knockdown EJ and UMUC3 cells after being infected with recombinant lentiviral vectors containing shRNAs targeting *DNMT3B* (shRNA) and negative control cells infected with empty lentivirus (NC). (**D**) The protein levels of HNF4γ and Notch1 in the shRNA groups of EJ and UMUC3 cells transfected with the miR-34a inhibitor (shRNA+inhibitor) or miR-NC (shRNA+miR-NC). NC cells were used as the control. DNMT3B, DNA methyltransferase 3B; HNF4γ, hepatocyte nuclear factor 4 gamma; UTR, untranslated region; NC, negative control; shRNA, short hairpin RNA.

To further investigate the underlying mechanisms of DNMT3B, we examined the expression of the downstream targets of miR-34a. Several studies have indicated that miR-34a modulates the migration and invasion of tumor cells by down-regulating hepatocyte nuclear factor 4 gamma (HNF4γ) and Notch1 [14–16]. As shown in Figure 1A, miR-34a has binding sites on the 3’ untranslated region (UTR) of HNF4γ and Notch1 mRNAs. miR-34a can downregulate the protein levels of HNF4γ and Notch1 in bladder cancer and endometrial cancer cells [14–16]. In this study, we further confirmed their targeted regulatory pathways in EJ and UMUC3 cells. The protein levels of HNF4γ and Notch1 were decreased in miR-34a-mimic transfected cells and increased in miR-34a-inhibitor transfected cells. These results indicate that HNF4γ and Notch1 are downstream targets of miR-34a in EJ and UMUC3 cells (Figure 6B). As shown in Figure 6C, the protein levels of HNF4γ and Notch1 were lower in the shRNA groups of EJ and UMUC3 cells compared to those in the NC groups, indicating that DNMT3B knockdown could decrease the protein levels of HNF4γ and Notch1 protein levels. Moreover, the protein levels of HNF4γ and Notch1 were higher in the shRNA groups of EJ and UMUC3 cells transfected with miR-34a inhibitor, compared to those transfected with miR-NC (Figure 6D), indicating that inhibiting miR-34a attenuated the inhibitory effects of *DNMT3B* knockdown on the downstream targets of miR-34a.

## DISCUSSION

In the present study, we investigated the clinical significance of DNMT3B expression and the underlying mechanisms of DNMT3B in promoting the migration and invasion of bladder cancer. Previous studies have indicated that DNMT3B is upregulated in various types of cancer [3, 17–19], and DNMT3B overexpression is involved in gastric tumorigenesis [3]. We examined 30 bladder cancer tissues and the level of DNMT3B expression was high in 20 tissues and low in 10 tissues. DNMT3B expression was not correlated with gender, age, or tumor size. However, it was correlated with node metastasis, T stages, and the muscle-invasive stage. Compared to patients with higher expression of DNMT3B, patients with lower expression of DNMT3B had a better prognosis, which is consistent with previous research in hepatocellular carcinoma [20]. These results indicate that DNMT3B may play a significant role in the tumor progression of bladder cancer. Therefore, it is necessary to explore the mechanisms of its regulatory functions.

As a DNA methyltransferase, DNMT3B has been involved in tumor progression by modulating the methylation of gene promoters important for proliferation, migration, and invasion [21–23]. For example, DNMT3B-mediated methylation of the metastasis suppressor 1 (MTSS1) promoter epigenetically suppresses MTSS1 transcription in the urothelial carcinoma of the bladder [23]. As important regulators of tumor progression and metastasis, the levels of many miRNAs are aberrant in bladder cancer tissues compared to normal tissues [24]. Therefore, it is important to identify the regulator of microRNA expression to explore the functions of these miRNAs. MiR-34a is one of the few miRNAs whose promoter regions are known. In addition, the expression of miR-34a is lower in tumors with advanced clinical stages and high histological grades than those with lower stages or grades. Functional assays *in vitro* and *in vivo* have showed that miR-34a acts as a tumor suppressor in bladder cancer [10, 11, 14, 16]. For example, a miR-34a inhibitor enhanced the migration ability of the bladder cancer cell line BIU-87 [25]. Furthermore, aberrant CpG methylation in the miR-34a promoter results in decreased miR-34a expression in several types of cancer, including bladder cancer, breast cancer, lung cancer, colon cancer, pancreatic cancer, ovarian cancer, and laryngeal squamous cell carcinoma [12]. Promoter hypermethylation of miR-34a is associated with poor overall survival and contributes to the metastasis and progression of laryngeal squamous cell carcinoma [26]. Importantly, patients with lower levels of DNMT3B expression had a better prognosis, while patients with lower levels of miR-34a expression had a poorer prognosis. Since the mechanism of its epigenetic regulation is unclear, miR-34a was chosen as the focus of this study. Aberrant promoter hypermethylation can downregulate tumor suppressor genes, thus leading to tumor initiation and progression. Therefore, we hypothesized that DNMT3B increased the methylation of the miR-34a promoter to epigenetically suppress miR-34a transcription.

To verify this hypothesis, we first analyzed the correlation between DNMT3B and miR-34a in bladder cancer tissues. Our data revealed that the level of miR-34a was negatively correlated with the level of DNMT3B. In addition, the methylation ratio of the miR-34a promoter was positively correlated with the level of DNMT3B and negatively correlated with the level of miR-34a. These results indicate that DNMT3B might suppress miR-34a expression by increasing the methylation ratio of the miR-34a promoter. *DNMT3B* overexpression in BIU-87 cells decreased miR-34a expression, increased miR-34a promoter methylation, and decreased the transcriptional activity of the miR-34a promoter, which also support our hypothesis. In addition, *DNMT3B* knockdown increased miR-34a expression, decreased miR-34a promoter methylation, and increased the transcriptional activity of the miR-34a promoter in both EJ and UMUC3 cells. All these results demonstrate that DNMT3B silencing decreases the methylation of the miR-34a promoter *in vitro*, resulting in the enhancement of miR-34a transcription. These results also support our above hypothesis.

We also verified whether DNMT3B was involved in the migration and invasion of bladder cancer via miR-34a. Since the role of DNMT3B in bladder cancer cell lines EJ and UMUC3 was undefined, we studied the effect of DNMT3B knockdown on the migration, invasion, and EMT in EJ and UMUC3 cells. Our results showed that DNMT3B knockdown inhibited the migration, invasion, and EMT in EJ and UMUC3 cells, while the miR-34a inhibitor rescued the inhibitory effects of *DNMT3B* knockdown on migration and invasion in EJ and UMUC3 cells. The miR-34a inhibitor can also promote the migration and invasion of EJ and UMUC3 cells, consistent with previous report about the effect of a miR-34a inhibitor in the bladder cancer cell line BIU-87 [25]. Our present results suggest that DNMT3B silencing suppresses migration and invasion by upregulating miR-34a in bladder cancer.

miRNAs play their roles primarily by inhibiting the translation of target gene mRNAs. Therefore, identifying the downstream targets of miR-34a is important to fully clarify the regulatory mechanism of DNMT3B. miR-34a has binding sites on the 3’ UTR of HNF4γ and Notch1 mRNAs, which have been identified as the direct targets of miR-34a [14, 15, 27]. Our results showed that miR-34a overexpression decreased the protein levels of HNF4γ and Notch1, supporting this observation. We have also found that DNMT3B knockdown could decrease protein levels of HNF4γ and Notch1 while the miR-34a inhibitor attenuated the inhibitory effects of *DNMT3B* knockdown on these downstream targets of miR-34a. All our results demonstrate that DNMT3B silencing increases HNF4γ and Notch1 protein via suppressing miR-34a expression. Several studies have indicated that miR-34a suppressed the migration and invasion of tumor cells by down-regulating HNF4γ and Notch1 [14–16], consistent with our hypothesis that DNMT3B may play its role by miR-34a-HNF4γ and/or Notch1 axis.

We also explored the relationship between EMT and DNMT3B. *DNMT3B* knockdown suppressed the expression of mesenchymal markers vimentin, N-cadherin, Snail, TWIST1 and ZEB1, and increased the expression of an epithelial marker E-cadherin [28]. Moreover, DNMT3B knockdown suppressed the expression of MMP2 and MMP9, which could induce the EMT process [29]. These results indicate that *DNMT3B* knockdown suppresses the EMT process in bladder cancer cell lines. Additionally, we found that the miR-34a inhibitor attenuated the inhibitory effects of *DNMT3B* knockdown on EMT, indicating that DNMT3B silencing suppresses EMT by upregulating miR-34a in bladder cancer. Notch1 is a downstream target of miR-34a and plays crucial roles in the EMT process. Since DNMT3B silencing increased Notch1 protein via suppressing miR-34a expression, we hypothesized that DNMT3B may promote EMT via the miR-34a-Notch1 axis. Since EMT promotes cancer cell migration and invasion [28], the suppression of miR-34a-Notch1 axis-induced EMT may be the regulatory mechanism of DNMT3B knockdown in suppressing the migration and invasion in bladder cancer.

Our study has some limitations. Firstly, only 30 bladder cancer tissues were available to assess the expression of miR-34a, which may be too small to support our conclusion. However, since the observed differences were statistically significant, the impact of the sample size on our findings may be limited. Secondly, although our results demonstrate that *DNMT3B* knockdown could decrease the protein levels of HNF4γ and Notch1 via upregulating miR-34a, we did not directly illustrate whether DNMT3B functions through HNF4γ and/or Notch1, which is important for understanding the regulatory mechanism of DNMT3B and will be the focus of our future studies.

In conclusion, our results have showed that the level of DNMT3B is positively correlated with the methylation ratio of the miR-34a promoter while DNMT3B silencing suppresses migration and invasion by epigenetically promoting miR-34a in bladder cancer cell lines. Increased level of miR-34a suppresses the protein level of Notch1, which is the downstream target of miR-34a, thereby suppressing EMT-induced migration and invasion. This may be the underlying mechanism of DNMT3B’s regulatory effect. Our findings reinforce the importance of DNMT3B in bladder cancer and demonstrate that DNMT3B could be a therapeutic target for bladder cancer.

## MATERIALS AND METHODS

### Patient samples and tissue microarray assay

Bladder cancer tissues (n=30) were obtained from the Zhujiang Hospital of Southern Medical University between 2017 and 2018. Informed consent was obtained from each patient. This study was approved by the ethics committee of the Zhujiang Hospital of Southern Medical University.

Microarray analysis was performed on all tissue samples. The level of DNMT3B was assessed by immunohistochemistry (IHC) (Shanghai Outdo Biotech Co., LTD.), and the DNMT3B-stained tissues were scored independently by two senior pathologists according to the standard protocol [30]. The total scores were divided into two categories: a score between 1 and 4 was considered low expression, and a score between 5 and 12 was considered high expression.

### BSP analysis

The genomic DNA of bladder cancer tissues and cells was extracted using the SolPure Cell DNA Kit (Magen). Bisulfite modification was carried out using an EpiTect Bisulfite Kit (Qiagen). The promoter region of miR-34a was amplified using HotStarTaq reagents (Qiagen) with 5'-GTTTTTGGTTTTAGAAGTTTTTT-3' and 5'-AATCCTAATCCTCATCCCCTTC-3' primers and cloned into a pGEM-T vector. Individual clones (n=10) from each group were sequenced and analyzed quantitatively using the online software BISMA.

### Cell culture

Human bladder cancer cell lines T24, BIU-87, EJ, and UMUC3 were obtained from the American Type Culture Collection. All cells were cultured in the RPMI 1640 medium containing 10% fetal bovine serum, 100 U/mL penicillin G, and 100 μg/mL streptomycin, and maintained at 37 °C in a humidified 5% CO_2_ incubator.

### Construction of *DNMT3B* shRNA stable knockdown cells

To construct the lentiviral vector containing the shRNA targeting *DNMT3B* (shRNA), the shRNA was amplified and inserted into a pLVX-shRNA2-Puro vector (Invitrogen). The sequence of the shRNA targeting *DNMT3B* was: 5'-AATTAAAAAAAGATGACGGATGCCTAGAGTCTCTTGAACTCTAGGCATCCGTCATCTCG -3'. EJ and UMUC3 cells were infected by either empty lentiviral vectors as the negative control (NC) or recombinant lentiviral vectors containing shRNAs targeting *DNMT3B* in the presence of 5 μg/mL polybrene (Sigma). Infected bladder cancer cells, namely NC or shRNA, were selected for 14 days using 2 μg/mL puromycin (Sigma). The knockdown of *DNMT3B* in infected bladder cancer cells was determined by qRT-PCR and western blot analyses.

### qRT-PCR

Total RNA was extracted using the TRIzol reagent (Ambion) according to the manufacturer’s instructions. RQ1 RNase-Free DNase (Promega) was used for the removal of contaminating DNA. To determine the expression of *DNMT3B* and miR-34a, complementary DNA was obtained by reverse transcription of total RNA using M-MLV reverse transcriptase (Promega). The reverse transcription primer for *DNMT3B* detection was oligo(dT). The reverse transcription primer for miR-34a detection was designed based on the universal stem-loop primer method. The expression of *DNMT3B* and miR-34a was determined using SYBR Green qPCR SuperMix (Invitrogen). The relative expression of *DNMT3B* and miR-34a was calculated using the 2^-ΔΔCT^ method based on the expression of the internal references 18S rRNA or U6 snRNA. The qRT-PCR primers and reverse transcription primers for miR-34a and U6 are listed in Supplementary Table 1.

### Western blot analysis

Total proteins were extracted using the RIPA Lysis Buffer and subjected to sodium dodecyl sulfate-polyacrylamide gel electrophoresis (SDS-PAGE). The proteins were transferred to polyvinylidene fluoride membranes. After blocking with 5% non-fat milk, the membranes were incubated with the primary antibodies anti-vimentin, 1:1000; anti-E-cadherin, 1:2000; anti-N-cadherin, 1:1000; anti-Snail, 1:2000; anti-TWIST1, 1:5000; anti-ZEB1, 1:1500; anti-MMP2, 1:1000; anti-MMP9, 1:2000; anti-HNF4γ, 1: 1000; anti-Notch1, 1:1500; and anti-GAPDH, 1:3000 (all antibodies were purchased from Abcam). The protein bands were visualized by incubating the membranes with the Immobilon Western Chemiluminescent HRP Substrate (Millipore Corporation, Billerica, MA, USA).

### Wound healing assay

Cells were seeded in six-well plates and cultured until confluent. A straight scratch was created using a pipette tip as previously described [31]. The wound healing at 0 h and 24 h were photographed using a LEICA microscope.

### Transwell assays

The Transwell assay was performed as previously described [32]. Bladder cancer cells were plated in the upper chamber. The cells at the topside of the filter membrane were removed using a cotton swab. The cells at the bottomside of the membrane were fixed with 4% paraformaldehyde and stained with 0.1% crystal violet solution. The migrated and invaded cells were monitored and photographed using a LEICA microscope at 400× magnification. Six randomly selected areas were counted. The experiments were repeated three times.

### Luciferase assays

miR-34a promotor luciferase reporter constructs were created by inserting the 1000 bp promoter region of miR-34a into a pGL3-Basic vector (Promega). The primers used for the miR-34a promotor were 5'-cggggtaccCCTTCCCACCCCAGCCCCCCGGGAC-3' and 5'-ccgctcgagCCCCGATCTGCGTGGTCACCGAGAA-3'. Cells were seeded in 24-well plates. The miR-34a promotor luciferase reporter plasmids and the internal control plasmids pRL-TK-Renilla (Promega) were co-transfected into the NC and shRNA groups of EJ and UMUC3 cells. The luciferase activity was examined 24 h after transfection using a Dual-Luciferase Assay Kit (Promega). The luminescence was examined using an Optima series luminometer.

### *DNMT3B-*overexpressing plasmid, miRNA mimic and cell transfection

The full coding sequence of *DNMT3B* was cloned into pcDNA3.1 plasmid by artificial DNA synthesis to construct the *DNMT3B-*overexpressing plasmid. The miR-34a inhibitor and miR-NC were purchased from GenePharma (Shanghai, China). The miR-34a inhibitor and miR-NC were transfected into *DNMT3B-*knockdown cells using Lipofectamine 2000 (Invitrogen) according to the standard protocol. The expression of miR-34a was determined using qRT-PCR to evaluate the transfection efficiency of the miR-34a inhibitor.

### Statistical analysis

Data were presented as means±standard deviations (SD). Statistical analysis was carried out using the SPSS 19.0 statistical analysis package (IBM Corporation, Armonk, NY, USA). The student’s *t*-test was performed to compare the effects of shRNA and NC on bladder cancer cells. The comparisons of more than two groups were conducted using one-way ANOVA followed by a post-hoc LSD test. Linear regression was performed for correlation analyses using GraphPad Prism version 7.0 (GraphPad Software, San Diego, CA, USA). Differences with *P*-values less than 0.05 were considered significant.

## Supplementary Material

Supplementary Figure 1

Supplementary Table 1
