# Supplementary material for: DNMT3B silencing suppresses migration and invasion by epigenetically promoting miR-34a in bladder cancer
Source: Aging (Albany NY). 2020 Nov 20;12(23):23668–83. doi: 10.18632/aging.103820 (PMC7762500; doi:10.18632/aging.103820)
Supplement: Supplementary Figure 1 [file aging-12-103820-s001.pdf]

SUPPLEMENTARY FIGURE

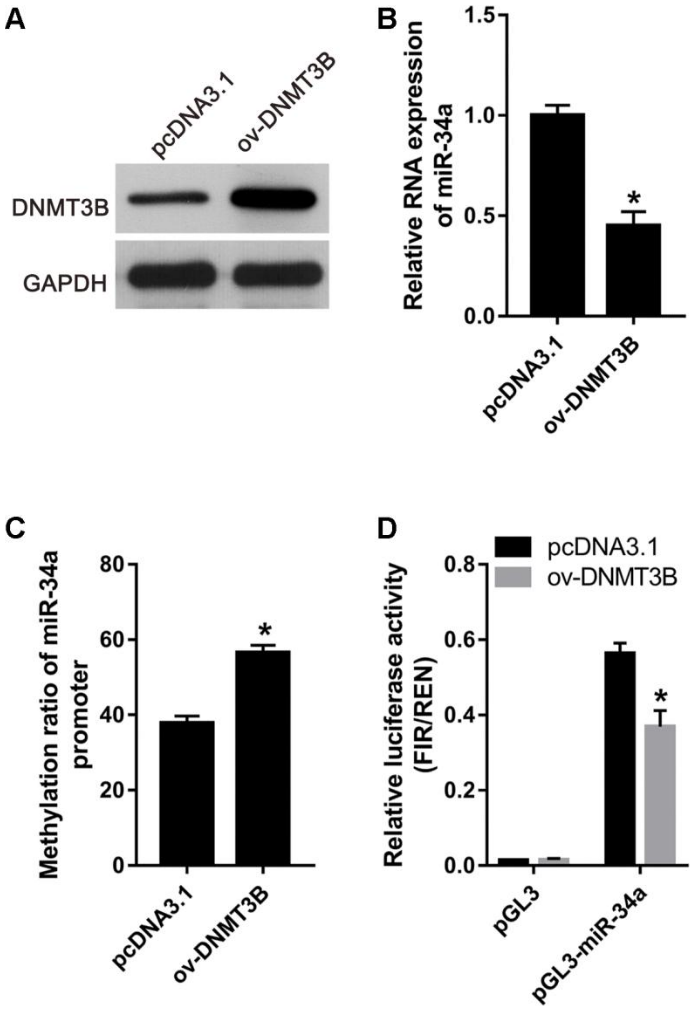

Supplementary Figure 1. The overexpression of *DNMT3B* decreased miR-34a expression and increased the methylation of the miR-34a promoter. *DNMT3B* overexpression plasmid (ov-DNMT3B) or empty plasmid pcDNA3.1 was transfected into BIU-87 cells. Forty-eight h later, cells were harvested and the expression of DNMT3B protein (**A**), miR-34a (**B**), and the methylation ratio of the miR-34a promoter (**C**) were determined by western blot (**A**), qRT-PCR (**B**), and bisulfite genomic sequencing (**C**), respectively. After transfected with ov-DNMT3B or pcDNA3.1 for 24 h later, a luciferase reporter plasmid containing the miR-34a promoter (pGL3-miR-34a) was transfected into BIU-87 cells to perform luciferase assays (**D**). The empty vector pGL3 was used as the control. The relative luciferase activity was calculated using the ratio of firefly and Renilla luciferase activities (**D**). Data were presented as means±SD. *p<0.05 between pcDNA3.1 and ov-DNMT3B. DNMT3B, DNA methyltransferase 3B; qRT-PCR, quantitative reverse transcription polymerase chain reaction; SD, standard deviation.
