# Supplementary material for: DNMT3B silencing suppresses migration and invasion by epigenetically promoting miR-34a in bladder cancer
Source: Aging (Albany NY). 2020 Nov 20;12(23):23668–83. doi: 10.18632/aging.103820 (PMC7762500; doi:10.18632/aging.103820)
Supplement: Supplementary Table 1 [file aging-12-103820-s002.pdf]

SUPPLEMENTARY TABLE

Supplementary Table 1. Primers used for qRT-PCR.

ID	sequence
miR-34a-F	ACACTCCAGCTGGGTGGCAGTGTCTTAGCTGGT
miR-34a-R	CTCAACTGGTGTCGTGGA
miR-34a-RT	CTCAACTGGTGTCGTGGAGTCGGCAATTCAGTTGAGACAACCAG
U6-F	CTCGCTTCGGCAGCACA
U6-R	AACGCTTCACGAATTTGCGT
U6-RT	AACGCTTCACGAATTTGCGT
HNF-4γ-F	TGCGTGTTCACAGCGGACCT
HNF-4γ-R	TGGCATTCACCGCGTGCCTT
NOTCH1-F	CATCTTGAGCACTAAGCCT
NOTCH1-R	GAGATAGACCAGTGGAGACA
8s-F	CCTGGATACCGCAGCTAGGA
18s-R	GCGGCGCAATACGAATGCCCC

F: forward primer; R: reverse primer; RT: reverse transcription primer.
